# Angle-Dependent Terahertz Circular Dichroism and Full-Space Polarization Manipulation via Extrinsic Chiral Metasurfaces

**DOI:** 10.3390/nano16100595

**Published:** 2026-05-13

**Authors:** Mengxiang Wan, Jiahao Shen, Hang Xu, Jialuo Ding, Cheng Chen, Qi Dong, Yuanyuan Lv, Lin Liu, Li Luo, Tingting Tang, Jie Li, Jianquan Yao

**Affiliations:** 1 Sichuan Province Key Laboratory of Optoelectronic Sensor Devices and Systems, College of Optoelectronic Engineering (Chengdu IC Valley Industrial College), Chengdu University of Information Technology, Chengdu 610225, China; wanmx1123@163.com (M.W.); 18628281335@163.com (J.S.); dingjialuo0324@163.com (J.D.); 18683371909@163.com (C.C.); dongqi@cuit.edu.cn (Q.D.); lyyuan01@163.com (Y.L.); liulinz60@163.com (L.L.); rolly80218@163.com (L.L.); skottt@163.com (T.T.); 2 Sichuan Meteorological Optoelectronic Sensor Technology and Application Engineering Research Center, Chengdu University of Information Technology, Chengdu 610225, China; 3 School of Precision Instruments and Opto-Electronics Engineering, Tianjin University, Tianjin 300072, China; xh_931119@tju.edu.cn (H.X.); jqyao@tju.edu.cn (J.Y.)

**Keywords:** terahertz waves, metasurface, polarization beam splitting, extrinsic chirality

## Abstract

Extrinsic chiral metasurfaces offer a promising route for controlling chiroptical responses through incident angle variation, yet the simultaneous realization of strong circular dichroism and full-space polarization beam splitting remains challenging. In this work, we propose an all-dielectric extrinsic chiral metasurface that leverages obliquely incident terahertz waves to break in-plane symmetry, thereby activating out-of-plane multipoles and inducing strong spin-selective scattering. At an incident angle of 30°, the metasurface achieves efficient full-space separation of left- and right-handed circularly polarized waves, with a circular dichroism peak exceeding 0.7 near 0.48 THz. Moreover, by varying the incident angle or operating frequency, the polarization state of the reflected wave can be continuously tuned from linear to elliptical to nearly circular, as visualized on the Poincaré sphere. This angle-dependent, full-space polarization manipulation capability highlights the potential of the proposed metasurface for applications in advanced terahertz imaging, LiDAR, and integrated photonic systems.

## 1. Introduction

Chirality, a geometric property describing an object that cannot be superimposed onto its mirror image, is ubiquitous in nature, ranging from the helical structure of DNA to the spiral arms of galaxies [1]. In optics, chirality manifests as chiroptical effects, including circular dichroism (CD) and optical activity (OA), which arise from the distinct interactions of left-handed circularly polarized (LCP) and right-handed circularly polarized (RCP) light with chiral media [2,3]. These phenomena have enabled a wide range of applications, from chiroptical spectroscopy and enantiomer-selective sensing to advanced imaging and quantum optics [4,5].

Metasurfaces, composed of planar subwavelength nanostructures, have emerged as a powerful platform for manipulating light-matter interactions, enabling the realization of chiroptical effects with strengths orders of magnitude greater than those found in natural materials [6,7,8,9,10,11]. Both intrinsic chirality, originating from the geometric asymmetry of the meta-atoms themselves [12,13], and extrinsic chirality, which arises from the symmetry breaking between the meta-atom and the illumination direction [14,15,16,17], have been extensively explored. In particular, extrinsic chirality introduces an additional degree of freedom-the angle of incidence-for controlling chiroptical responses, thereby enriching the toolkit for applications such as spin-selective wavefront shaping, chiral imaging, and ultrasensitive detection [18].

Beyond dichroism, metasurfaces have demonstrated remarkable capabilities in polarization beam splitting and polarization transformation [19,20]. Polarization beam splitters, which separate orthogonal polarization states into distinct spatial channels, can be categorized into half-space devices (both reflected or both transmitted beams) and full-space devices (one reflected and one transmitted beam). They can be applied to advanced LiDAR and optical imaging technologies [21,22]. Unequal power splitting between orthogonal components can be regarded as a form of polarization conversion, transforming an incident wave into a reflected or transmitted wave with a different polarization state [23]. While phase-gradient metasurfaces employing anisotropic meta-atoms offer a flexible and broadband approach to achieve beam splitting, this method primarily yields half-space separation and is inherently local [24,25,26]. An alternative strategy utilizes phase interference between multiple meta-atoms within a supercell to realize full-space beam splitting, but this comes at the cost of increased design complexity and a larger device footprint [27,28]. More recently, the intrinsic far-field scattering properties of individual meta-atoms have been harnessed to achieve nonlocal linear or circular polarization beam splitting [29]. For circular polarization, chiral metasurfaces provide a direct and powerful means to separate LCP and RCP components in reflection and transmission spaces [16,30]. However, the exploration of circular polarization beam splitters with an explicit and controllable angular dependence (full-space), a direct consequence of extrinsic chirality, remains relatively underexplored, promising a new dimension for advanced polarization control in integrated photonic systems.

In this work, we propose an all-dielectric extrinsic chiral metasurface that enables angle-dependent CD and full-space polarization manipulation in the terahertz regime. By introducing oblique incidence to break the in-plane symmetry of the U-shaped meta-atoms, we systematically investigate the underlying multipolar mechanisms that govern the spin-selective scattering response. Through full-wave simulations, we analyze the CD spectra, polarization beam splitting performance, and polarization conversion characteristics of the metasurface as functions of incident angle and operating frequency. This U-shaped, intrinsically achiral geometry was chosen to purely harness extrinsic chirality, where oblique incidence activates out-of-plane multipoles that yield switchable circular dichroism. Compared to intrinsically chiral or supercell-based designs, its key advantage is achieving high energy utilization efficiency, full-space circular polarization beam splitting and tunable polarization conversion with a single, simple dielectric resonator. The results demonstrate a versatile platform capable of achieving tunable chiroptical effects and comprehensive polarization control, paving the way for advanced terahertz photonic applications.

## 2. Results and Discussion

**Figure 1:**
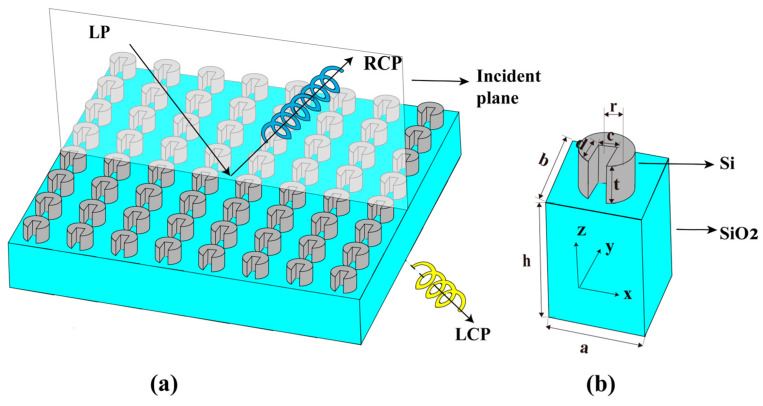
Schematic diagram of the function and structure of the extrinsic chiral metasurface: (**a**) angle-dependent circularly polarized terahertz wave beam splitting function; (**b**) Schematic diagram of the structure and dimensions of the meta-atom.

Figure 1 illustrates the proposed extrinsic chiral metasurface, including its functional principle and the structure of the constituent meta-atom. In Figure 1a, a linearly polarized terahertz wave is incident obliquely on the all-dielectric metasurface in the xoz plane at an angle θ. Due to extrinsic chirality, part of the incident wave is converted into circularly polarized components, which are then either reflected or transmitted. The extent of separation between the orthogonal circular polarizations depends on both the incidence angle and the operating frequency. At appropriate angles and frequencies, the reflected RCP wave and LCP wave exhibit comparable amplitudes with high polarization conversion efficiency. In this regime, the metasurface functions as a circular polarization beam splitter. Under most other conditions, however, the amplitudes of the reflected and transmitted waves become unbalanced, and the metasurface instead acts as a polarization converter, transforming the incident linearly polarized wave into an elliptically polarized one. Figure 1b presents the geometry of a single all-dielectric meta-atom, where the gray resonator is made of high-resistivity silicon and the blue substrate is silica. The resonator features a U-shaped structure, possessing only one mirror symmetry plane along the yoz direction. The optimization followed a coarse-to-fine strategy. We first empirically chose a reasonable initial resonator height, set the simulation frequency range to 0.1–1 THz, and fixed the incident angle at 30°. The U-shaped meta-atom was formed by subtracting a cylinder from a cube, and the cylinder radius as well as the cube’s length and width were coarsely swept to obtain a strong CD response. Once a pronounced CD peak appeared, we narrowed the frequency window and performed finer parametric optimization to finalize the geometry.

Here we explore the chiral response of the proposed structure. Considering an anisotropic meta-atom, and taking the reflected wave as an example, the relationship between its circular polarization scattering matrix and its linear polarization counterpart can be expressed as

(1) $$\left(\begin{array}{cc} R_{RR} & R_{RL}\\ R_{LR} & R_{LL} \end{array}\right)=\frac{1}{2}\left(\begin{array}{cc} R_{xx}+R_{yy}+i(R_{xy}-R_{yx}) & R_{xx}-R_{yy}-i(R_{xy}+R_{yx})\\ R_{xx}-R_{yy}+i(R_{xy}+R_{yx}) & R_{xx}+R_{yy}-i(R_{xy}-R_{yx}) \end{array}\right)$$

where the first and second subscripts denote the outgoing and incident wave components, respectively. The reflective CD spectra of the metasurface at specific incident angles can be calculated as

(2) $$\mathrm{CD}=\left({\left|R_{RL}\right|}^{2}+{\left|R_{LL}\right|}^{2}\right)-\left({\left|R_{LR}\right|}^{2}+{\left|R_{RR}\right|}^{2}\right).$$

We performed the simulations using CST Microwave Studio. The lateral boundaries of the meta-atom (in the xoy plane) were set to unit cell boundary conditions (the wave source is Floquet mode), while the longitudinal direction (z-axis) was set to open boundaries. The numerical grid size is “10 cells per max model box edge”. During the simulation process, the refractive index of high-resistivity silicon is n_1_ = 3.48, and that of silica is n_2_ = 2.14, where the dispersion can be neglected. Based on the above optimization strategy, the key geometric parameters are as follows. The lattice constants are a = b = 300 μm, the substrate thickness is h = 500 μm, the in-plane dimensions of the resonator are d = 200 μm and c = 60 μm, the corner radius is r = 140 μm, and the resonator height is t = 100 μm.

**Figure 2:**
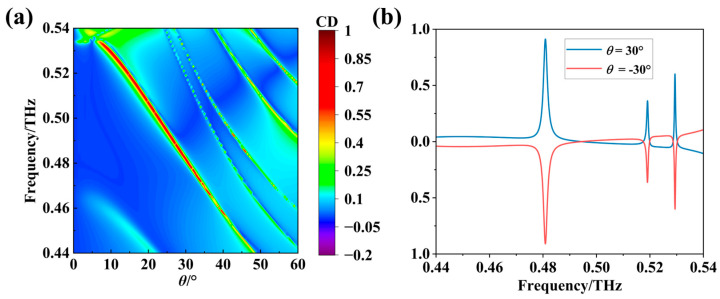
Angle-dependent characteristics of the reflective CD of the metasurface: (**a**) CD spectra for incident angles ranging from 0° to 60°; (**b**) comparison of CD curves at incident angles of 30° and −30°.

Figure 2a presents the frequency-angle two-dimensional distribution of the CD as the incident angle varies from 0° to 60°. As the incident angle changes, the CD peak exhibits a distinct frequency shift: smaller angles correspond to higher optimal operating frequencies. Notably, within the incident angle range of 10° to 35°, CD peak values exceeding 0.7 can be achieved across nearly the entire range, indicating that the metasurface possesses excellent polarization beam-splitting performance and exhibits significant robustness to variations in the incident angle. Figure 2b shows the CD spectra at incident angles of 30 degrees and −30 degrees. It can be observed that the CD spectra exhibit opposite values when the incident angles are opposite in sign. This behavior arises because the metasurface demonstrates distinct polarization conversion and beam-splitting functionalities when the incident wave propagates along symmetric directions. Specifically, at θ = −30°, the metasurface converts most of the incident LCP waves into transmitted RCP waves, while simultaneously converting incident RCP waves into reflected LCP waves. This corresponds to R_RL_ > R_LR_, which is entirely opposite to the case at θ = 30°, where R_RL_ < R_LR_.

**Figure 3:**
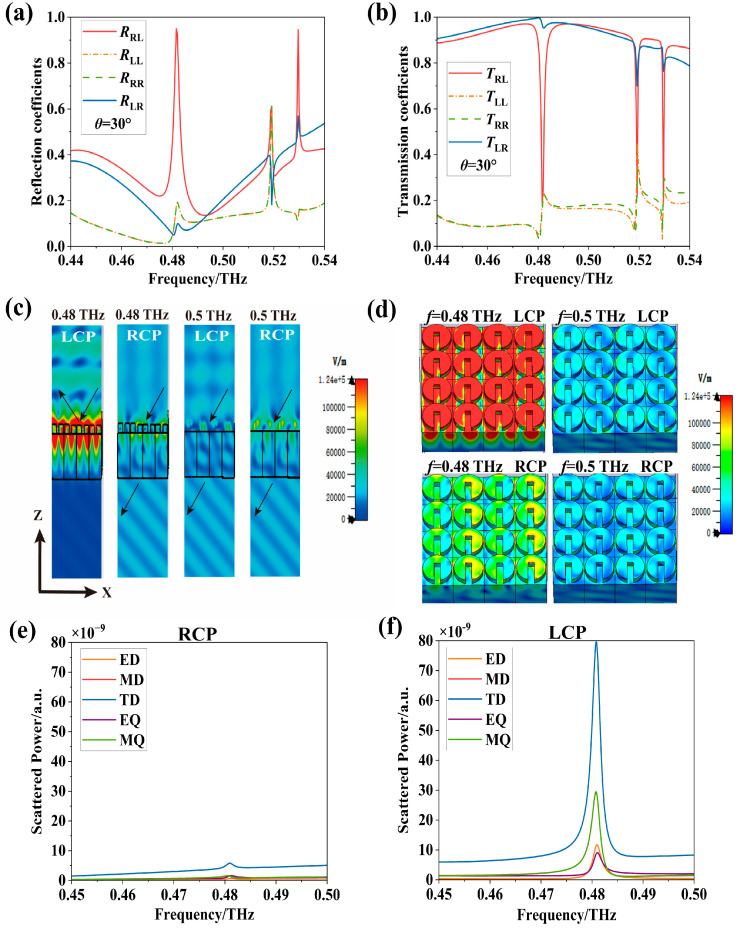
Analysis of the extrinsic chiral response and polarization beam-splitting functionality of the metasurface (at an incident angle θ = 30°): (**a**) circularly polarized reflection coefficients; (**b**) circularly polarized transmission coefficients; (**c**) demonstration of circular polarization beam-splitting functionality: comparison of reflected and transmitted electric fields at operating frequencies f = 0.48 THz and f = 0.5 THz; (**d**) analysis of local electric fields of meta-atoms at operating frequencies f = 0.48 THz and f = 0.5 THz; (**e**,**f**) multipole decomposition under LCP and RCP wave excitation.

Utilizing the optimized geometrical parameters of the meta-atom obtained above, we proceed to analyze the polarization manipulation capabilities of the metasurface. Figure 3a,b present the circularly polarized reflection and transmission coefficients of the device at an incident angle of θ = 30°. Given the absence of electromagnetic loss in the resonator, the reflection and transmission coefficients vary in inverse proportion: when R_RL_ reaches its maximum, T_RL_ drops to its minimum. Across the entire frequency range, the primary peak appears near 0.48 THz (the operating frequency of interest), with two additional extrema emerging around 0.52 THz and 0.53 THz. Notably, near the operating frequency, the LCP-to-RCP reflection component (R_RL_) dominates over other reflection coefficients, while in transmission, the RCP-to-LCP component (T_RL_) is significantly larger than the others. This indicates that the metasurface converts most of the incident LCP waves into reflected RCP waves and simultaneously converts incident RCP waves into transmitted LCP waves, achieving full-space separation of orthogonal circularly polarized beams. From an experimental feasibility standpoint, the resonant response does exhibit a moderately high Q-factor, but the required source bandwidth is readily met by a high-resolution (about 0.58 GHz) ZnTe terahertz time-domain spectroscopy (THz-TDS) system [31], which typically covers 0.3–0.8 THz or more and thus naturally encompasses our operating range (e.g., 0.44–0.54 THz for polarization-state tuning). Of course, a higher-resolution (about 140 MHz) terahertz frequency-domain spectrometer may also be used [32].

To provide a more intuitive demonstration of this functionality, Figure 3c illustrates the electric field distributions for different incident polarizations at two distinct operating frequencies. At f = 0.48 THz, an incident LCP wave excites a strong electromagnetic resonance near the meta-atoms, leading to dominant reflection, whereas under the same conditions, an incident RCP wave induces relatively weak resonance, resulting in high transmission efficiency. In contrast, at f = 0.50 THz, neither circular polarization state excites a strong resonance, and both exhibit high transmission. This phenomenon is further illustrated in Figure 3d from the perspective of the xoy plane, where an order-of-magnitude difference in the induced electric field intensity can be observed between the two operating frequencies. The CD and polarization beam-splitting phenomena can be explained as follows. Under oblique incidence, the longitudinal field component introduced by the terahertz wave vector disrupts the in-plane symmetry of the system, thereby effectively activating out-of-plane electric and magnetic dipole moments that remain unexcited or canceled under normal incidence. These out-of-plane multipoles engage in strong near-field coupling and interference with the dominant in-plane dipole moments, producing interference terms that contribute with opposite signs to LCP and RCP light. This ultimately manifests as a pronounced extrinsic chiral CD response at the level of multipolar scattering. Figure 3e,f show the multipole decomposition under LCP and RCP wave excitations, indicating that the TD (Toroidal Dipole) excited by LCP waves is much stronger than that excited by RCP waves, leading to circular dichroism.

**Figure 4:**
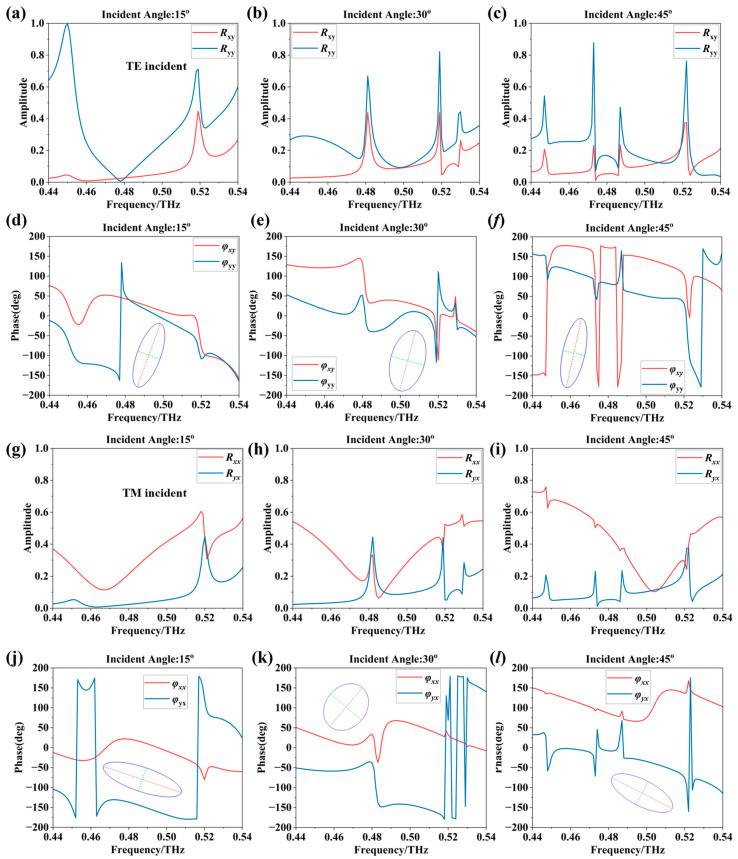
Polarization conversion performance of the metasurface: (**a**–**c**) reflection amplitudes of TE-polarized waves at incident angles θ = 15°, 30°, and 45°; (**d**–**f**) corresponding reflection phases and polarization ellipses at 0.48 THz. (**g**–**i**) reflection amplitudes of TM-polarized waves at incident angles θ = 15°, 30°, and 45°; (**j**–**l**) corresponding reflection phases and polarization ellipses at 0.48 THz.

Given the significant extrinsic chiral response exhibited by the proposed metasurface, a potential application-angle-dependent polarization conversion is demonstrated here, including TE- and TM-polarized incident waves. For convenience, we denote the TE and TM polarization states of the incident wave as x and y polarization, respectively. Figure 4a–c illustrate the reflection amplitudes for TE-polarized waves at incident angles θ = 15°, 30°, and 45°, where R_xy_ and R_yy_ represent the co- and cross-polarized components of the TE wave, respectively. The corresponding reflection phases are shown in Figure 4d–f. With increasing incident angle, both the amplitude and phase of the orthogonal polarization component undergo significant variations, accompanied by a shift in the optimal operating frequency. Notably, at an incident angle of 30°, the reflection coefficients of the two polarization components approach approximately 0.5 near f = 0.48 THz, resulting in an elliptically polarized state with a large ellipticity, approaching circular polarization. Similarly, Figure 4g–j show the case of TM-polarized wave incidence.

**Figure 5:**
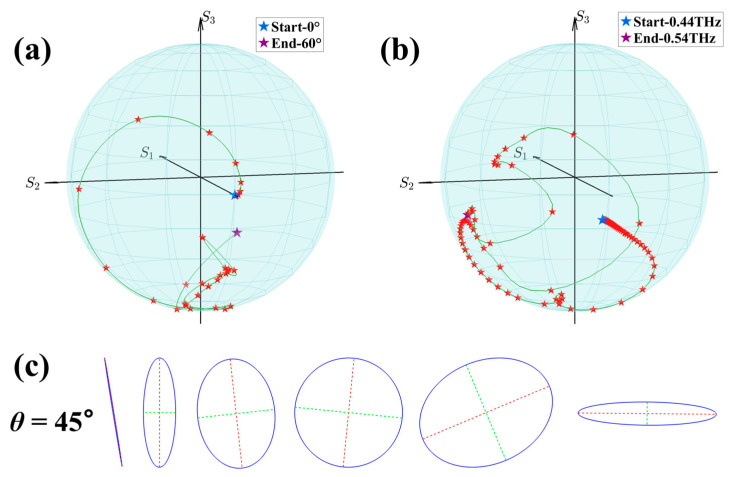
Coverage of the polarization conversion functionality of the metasurface on the Poincaré sphere: (**a**) variation in polarization states at different incident angles; (**b**) polarization conversion at different frequencies for an incident angle θ = 30°; (**c**) polarization ellipses at different frequencies for an incident angle θ = 45°.

To further illustrate the polarization conversion performance of the proposed metasurface for linearly polarized incident waves, we present the polarization states of the reflected waves using the Poincaré sphere. Figure 5a shows the polarization states at f = 0.48 THz as the incident angle increases from 0° to 60°. It can be observed that the evolution trajectory of the polarization states spans a wide range, covering linear, elliptical, and nearly circular polarization states. Figure 5b depicts the polarization states for incident angle θ = 30° within the frequency range of 0.44–0.54 THz. Similarly, the evolution of the reflected waves on the Poincaré sphere encompasses various polarization types. Figure 5c presents the polarization ellipses of the reflected waves at several distinct frequencies under an incident angle of θ = 45°. Significant variations in both ellipticity and orientation angle can be observed, indicating that the proposed metasurface is capable of imparting a wide variety of polarization states to linearly polarized incident waves. The polarization states presented in Figure 5 are mainly concentrated near the south pole of the Poincaré sphere, which is due to the chosen incident angle. If the incident angle is changed to −30°, these polarization states will flip and become concentrated near the north pole. On the other hand, the slight deviation of the polarization states from the south poles (perfect circular polarization) arises mainly from the amplitude imbalance and the phase deviation between the two orthogonal reflected field components. The detailed optimal performance parameters for polarization conversion are shown in Table 1.

We have listed several representative references in Table 2 and compared them with this work. Overall, the results of this work exhibit competitive CD values, demonstrate a wider range of incident angles, and notably, operate in the terahertz frequency band.

## 3. Conclusions

In conclusion, we have theoretically and numerically demonstrated an all-dielectric extrinsic chiral metasurface that achieves angle-tunable CD and full-space polarization beam splitting in the terahertz regime. The design exploits oblique incidence to break the in-plane mirror symmetry of the U-shaped meta-atoms, thereby activating out-of-plane electric and magnetic multipoles that are otherwise suppressed under normal incidence. These out-of-plane multipoles couple with the dominant in-plane dipolar responses, giving rise to destructive interference terms that contribute oppositely for LCP and RCP waves, which ultimately manifests as a pronounced and switchable chiral response. By tailoring the incident angle, the metasurface enables efficient full-space separation of orthogonal circular polarizations, with a CD exceeding 0.7 near 0.48 THz, while simultaneously allowing continuous tuning of the reflected polarization state from linear to elliptical to nearly circular across a broad angular and frequency range. Unlike conventional half-space polarization splitters or designs relying on complex supercell engineering, our approach achieves nonlocal, angle-dependent full-space beam splitting using a single-layer passive structure, combining design simplicity with functional versatility. This work establishes a systematic route for harnessing extrinsic chirality to achieve integrated control over CD, beam splitting, and polarization conversion, offering promising potential for applications in terahertz imaging, spin-selective communication, and compact photonic systems.

## Figures and Tables

**Table 1:** Performance parameters of polarization conversion.

*θ*	$E_{x}$	$E_{y}$	$\varphi_{x}$	$\varphi_{y}$	Ellipticity	Azimuth/°
30°	0.16464	0.22158	144.02226	50.08682	−0.7370	−84.5860
45°	0.12217	0.12361	135.71993	45.86258	−0.9881	84.0025

**Table 2:** Comparison of the core parameters.

References	CD/Extinction Ratio	Frequency/Wavelength	Incident Angle
Ref. [14]	CD > 6 dB	5–7 GHz	−30°\30°
Ref. [15]	\	1600 nm	−50–50°
Ref. [29]	Extinction ratio > 25 dB	~20 GHz	0–45°
This work	CD > 0.7	~0.48 THz	−60–60°

## Data Availability

The original contributions presented in this study are included in the article. Further inquiries can be directed to the corresponding author.
